# Wiskott-Aldrich Syndrome Protein: Roles in Signal Transduction in T Cells

**DOI:** 10.3389/fcell.2021.674572

**Published:** 2021-06-08

**Authors:** Jatuporn Ngoenkam, Pussadee Paensuwan, Piyamaporn Wipa, Wolfgang W. A. Schamel, Sutatip Pongcharoen

**Affiliations:** ^1^Department of Microbiology and Parasitology, Faculty of Medical Science, Naresuan University, Phitsanulok, Thailand; ^2^Department of Optometry, Faculty of Allied Health Sciences, Naresuan University, Phitsanulok, Thailand; ^3^Signalling Research Centers BIOSS and CIBSS, University of Freiburg, Freiburg, Germany; ^4^Department of Immunology, Faculty of Biology, University of Freiburg, Freiburg, Germany; ^5^Centre for Chronic Immunodeficiency (CCI), Freiburg University Clinics, University of Freiburg, Freiburg, Germany; ^6^Department of Medicine, Faculty of Medicine, Naresuan University, Phitsanulok, Thailand

**Keywords:** WASp, T cell signaling, T cell activation, MAPK, PKC, calcium

## Abstract

Signal transduction regulates the proper function of T cells in an immune response. Upon binding to its specific ligand associated with major histocompatibility complex (MHC) molecules on an antigen presenting cell, the T cell receptor (TCR) initiates intracellular signaling that leads to extensive actin polymerization. Wiskott-Aldrich syndrome protein (WASp) is one of the actin nucleation factors that is recruited to TCR microclusters, where it is activated and regulates actin network formation. Here we highlight the research that has focused on WASp-deficient T cells from both human and mice in TCR-mediated signal transduction. We discuss the role of WASp in proximal TCR signaling as well as in the Ras/Rac-MAPK (mitogen-activated protein kinase), PKC (protein kinase C) and Ca^2+^-mediated signaling pathways.

## Introduction

Wiskott-Aldrich syndrome (WAS) is a severe X-linked primary immunodeficiency caused by the mutations of the *WAS* gene on the X-chromosome. WAS is characterized by thrombocytopenia, eczema, increased susceptibility to infection and increased risk to develop autoimmune disease (Gallego et al., 1997; Bosticardo et al., 2009; Massaad et al., 2013). The *WAS* gene encodes the Wiskott-Aldrich syndrome protein (WASp), which is of the actin nucleation-promoting factor family. WASp is a cytosolic protein comprising 502 amino acids lacking intrinsic catalytic activity. Instead, WASp acts as scaffold protein that transduces a wide range of signals from cell surface receptors to mediate dynamic changes in the actin cytoskeleton in response to external stimuli (Sun et al., 2019). WASp is expressed exclusively in the hematopoietic cell lineages including T cells, B cells, natural killer (NK) cells, dendritic cells, macrophages, and platelets (Matalon et al., 2013). T cells isolated from WAS patient have a defect in actin reorganization in response to TCR-mediated stimulation (Gallego et al., 1997; Matalon et al., 2013). Furthermore, WASp has an essential role in signal transduction and effector functions of T cells.

Wiskott-Aldrich syndrome protein belongs to the WASp family of proteins consisting of WASp, neuronal (N)-WASp, and WAVE 1–3 and WASp and SCAR homolog (WASH) (Linardopoulou et al., 2007). Although, WASp and N-WASp share more than 50% sequence homology as well as having similar protein binding partners and basic functions, these two proteins are not entirely redundant (Fried et al., 2014). In contrast to WASp, N-WASp is widely expressed in multiple tissues (Miki et al., 1996).

In this article, we review recent findings of WASp in TCR-mediated signal transduction. Since the adaptor protein non-catalytic region of tyrosine kinase (Nck) binds to WASp, we discuss the mechanisms of Nck-mediated WASp recruitment to the TCR whereby it may regulate the actin machinery and signal transduction in close proximity to the TCR.

## WASp Structure and Its Change During T Cell Activation

**FIGURE 1:**
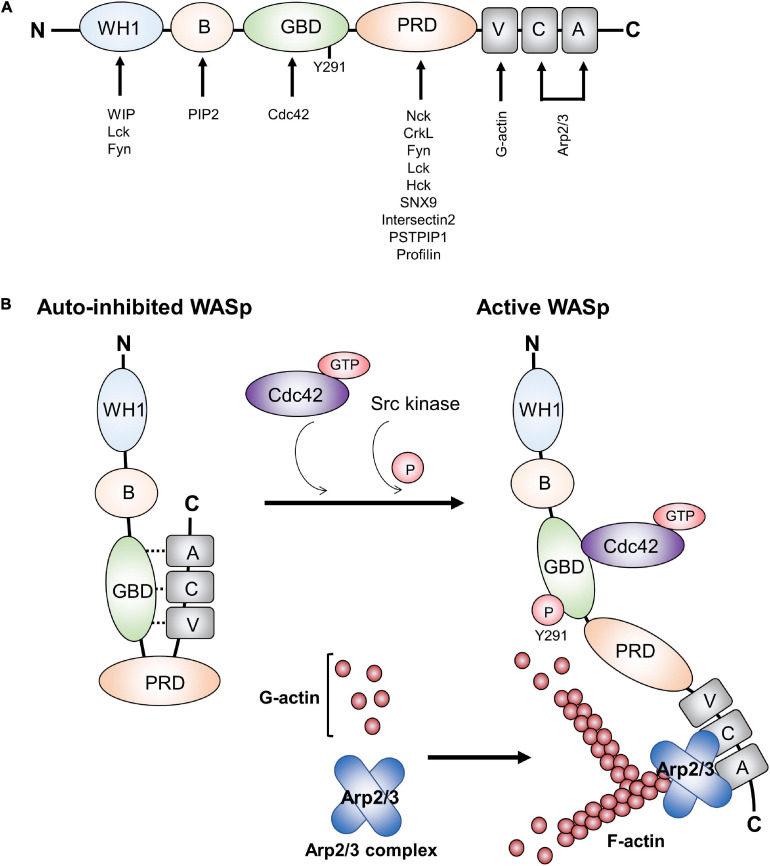
Functional domains in WASp and WASp activation. **(A)** WASp contains various domains, which can bind proteins involved in TCR-mediated actin cytoskeleton remodeling and signal transduction. **(B)** WASp is in a closed auto-inhibited conformation in resting state due to its intracellular interaction between GBD and VCA domains. Upon TCR engagement, it mediates the binding of GTP-Cdc42 to the GBD domain, thereby releasing the VCA domain from GBD domain and changing the WASp structure into an opened conformation. In addition, WASp tyrosine 291 within GBD can be phosphorylated by the Src family kinases Fyn and Lck. Recruitment of GTP-Cdc42 to GBD and phosphorylation of WASp tyrosine 291 within GBD result in WASp activation. Subsequently, Arp2/3 and monomeric actin can bind the VCA domain, which induces a new actin branch. This figure was modified from Matalon et al. (2013). Wiskott-Aldrich syndrome protein–dynamic regulation of actin homeostasis: from activation through function and signal termination in T lymphocytes. Immunol. Rev. 256(1), 10–29; with a permission from John Wiley and Sons; license number 5065241058992.

From the N- to the C-terminus, WASp contains a WASp homology 1 (WH1) domain (also known as EVH1, for ENA/VASP homology), a basic (B) domain, a GTPase-binding domain (GBD), a proline-rich domain (PRD), and a verprolin homology (V), cofilin (C) homology, and acidic region (A) (VCA domain) at the C-terminus (Zhang et al., 2009; Figure 1A). These distinct domains are required to mediate downstream signaling by binding to different cytoskeleton-regulating protein partners. More than 20 protein binding partners have been reported (Thrasher and Burns, 2010). Although, the structures of the WASp family members vary, the VCA domain is particularly conserved. The VCA domain interacts with actin and Arp2/3, whereas the PRD domain binds to various SH3 domain-containing proteins.

The first N-terminal WH1 domain is a binding site for a proline repeat motif present in the WASp interacting protein (WIP) (Volkman et al., 2002). WASp is constitutively associated with WIP. WIP regulates WASp activity and promotes WASp stability in resting T cell by protecting WASp from degradation by calpain and proteasome. It is also critical for localizing WASp to areas of actin polymerization (Chou et al., 2006; de la Fuente et al., 2007). In addition, the WH1 domain may act as a binding site for the Src family kinase Fyn and Lck in T cells (Sato et al., 2011; Matalon et al., 2013). Following the WH1 domain, the B domain is involved in the regulation of the WASp conformation, as it can bind the phosphoinositide PIP2 (phosphatidylinositol-4,5-bisphosphate) and acts in couple with the small GTPase Cdc42 to release of WASp from its auto-inhibited conformation toward the active conformation (Higgs and Pollard, 2000; Thrasher and Burns, 2010). The GBD domain can interact in *cis* with the C-terminal VCA domain, thereby inducing the closed autoinhibitory conformation (Figure 1B; Kim et al., 2000). Upon activation, the VCA domain is released from GBD as a consequence of the binding of GTP-bound Cdc42 to the WASp GBD. The PRD serves as the docking site for multiple protein binding partners that contain an SH3 domain such as Src and Tec family tyrosine kinases (Bunnell et al., 1996; Torres and Rosen, 2006) and the adaptor protein Nck (Rivera et al., 2004; Barda-Saad et al., 2005). Finally, after TCR engagement and the recruitment of GTP-bound Cdc42 to the WASp GBD, the released VCA region can interact with both monomeric actin (through the V region) and with the actin-related protein complex Arp2/3 (through the CA region) that work together to stimulate nucleation of branched actin filaments (Figure 1B; Symons et al., 1996; Miki and Takenawa, 1998; Blanchoin et al., 2000; Krause et al., 2000).

In resting T cells, WASp is mainly present in an autoinhibited conformation in the cytoplasm, in which the VCA domain interacts with a hydrophobic patch located within the GBD (Figure 1B). Upon TCR-mediated signaling, the kinase Zeta-associated protein of 70 kDa (ZAP-70) is recruited to the TCR and activated. Subsequently, it phosphorylates the adaptor protein Src homology 2 (SH2) domain-containing leukocyte protein of 76 kDa (SLP-76) that has a binding site for Nck and the guanine nucleotide exchange factor Vav-1. Nck is constitutively associated with WASp *via* the C-terminal SH3 domain of Nck binding to the PRD of WASp (Rivero-Lezcano et al., 1995; Paensuwan et al., 2015). Thus, Nck acts as a bridge to recruit WASp to the SLP-76 signaling complex. In association with SLP-76, Vav-1 mediates the exchange of GDP- to GTP-bound Cdc42, Rho family GTPases. GTP-bound Cdc42 then interacts with the WASp GBD, thereby releasing WASp from its auto-inhibited conformation, allowing VCA to bind to the Arp2/3 complex. Once bound to the VCA domain, Arp2/3 promotes the branching of the actin polymerization and rearrangement at the T cell-APC contact site (Zeng et al., 2003; Matalon et al., 2013). In addition, tyrosine 291 within WASp GBD can be phosphorylated by the Src family kinases Fyn and Lck (Badour et al., 2004; Torres and Rosen, 2006), which interact with the WASp WH1 domain (Sato et al., 2011). Phosphorylation of tyrosine 291 is essential for WASp activation (Cory et al., 2002; Badour et al., 2004). Thus, besides the recruitment of Cdc42 to WASp, WASp activation can be indicated by tyrosine 291 phosphorylation. Interestingly, phosphorylation of Y291 within WASp GBD domain also mediates WASp degradation by calpain and proteasome proteolysis (Watanabe et al., 2013; Sun et al., 2019).

## Initiation and Following Pathways of TCR Signaling

Following TCR engagement by its ligand peptide-MHC (pMHC), the TCR and its signaling molecules rapidly form microclusters where signaling is amplified and sustained (Seminario and Bunnell, 2008; Choudhuri and Dustin, 2010). The TCR is composed of the pMHC-binding TCRαβ heterodimer non-covalently associated with the non-variable signal transduction subunits CD3εγ, CD3εδ, and CD3ζζ (Kane et al., 2000; Alarcón et al., 2003). The TCRα and TCRβ chains have short cytoplasmic tails with no intrinsic capacity to mediate signal transduction. In contrast, the CD3 molecules serve as signal transducers by transferring information of TCR pMHC-binding to initiate signaling transduction (Hayes et al., 2003; Schamel et al., 2019). Each of the CD3 signaling subunits has cytoplasmic immunoreceptor tyrosine-based activation motifs (ITAMs) (Reth, 1989), one present in CD3ε, CD3δ, and CD3γ and three in CD3ζ. In addition, CD3ε has a proline-rich sequence (PRS) (Gil et al., 2002) and the receptor kinase (RK) motif (Hartl et al., 2020), which are required to regulate TCR activation. We will describe these two significant motifs in the next section.

T cell receptor engagement results in phosphorylation on tyrosine residues within the ITAMs, creating pairs of phosphotyrosines, which serve as docking sites for proteins containing SH2 domains such as ZAP-70. Following the binding of ZAP-70 to ITAMs, ZAP-70 phosphorylates LAT (linker for activation of T cells). Phosphorylated LAT recruits various enzymes and adaptor proteins to form multi-protein signaling complexes (Zhang et al., 1998). Recently, the formins mDia1 and mDia3 play a crucial role in F-actin polymerization, which facilitate LAT phosphorylation by ZAP-70 (Thumkeo et al., 2020). Phosphorylated LAT can bind to phospholipase Cγ1 (PLCγ1), phosphoinositide 3-kinase (PI3K), growth factor receptor-bound protein 2 (Grb2) and GRB2-related adaptor downstream of Shc (Gads). Gads serves as a bridge to recruit SLP-76 to phospho-LAT, where SLP-76 is phosphorylated by ZAP-70. As described above, WASp is recruited to SLP-76 through Nck, where WASp can associate and activate Arp2/3 to promote actin filament formation (Wunderlich et al., 1999; Barda-Saad et al., 2005).

After the signaling complexes have formed, they are required to activate four pathways: Ras/Rac-mitogen-activated protein kinase (MAPK), protein kinase C (PKC), nuclear factor of κB (NF-κB) and Ca^2+^-mediated signaling pathways, such as the nuclear factor of activated T cells (NFAT) pathway. The Ras pathway activates the extracellular receptor-activated kinase (Erk), a member of the MAP kinase family. The activated Erk translocates to the nucleus and phosphorylates its substrate Elk1. Phospho-Elk1 then stimulates the transcription of c-Fos, a component of the transcription factor the activation protein 1 (AP-1). In parallel with the Ras pathway, Rac is activated by Vav-1, thereby generating Rac-GTP. The active Rac-GTP activates another MAP kinase called c-Jun *N*-terminal kinase (Jnk). Once activation, Jnk then phosphorylates c-Jun, the second component of AP-1 (Smith-Garvin et al., 2009; Conley et al., 2016).

PLCγ1 bound to phospho-LAT is phosphorylated by ZAP-70 and the Tec family kinase Itk. Phosphorylated PLCγ1 catalyzes the hydrolysis of the plasma membrane phospholipid PIP2 generating two breakdown products, membrane-bound diacylglycerol (DAG) and inositol 1,4,5-trisphosphate (IP3) (Rhee and Bae, 1997; Braiman et al., 2006). DAG leads to the activation of PKCθ, which then mediates the activation and nuclear translocation of NF-κB. IP3 stimulates the increase in intracellular Ca^2+^, which subsequently activates the transcription factor NFAT to translocate to the nucleus. In the nucleus, the transcription factors AP-1, NF-κB and NFAT bind to promotors of specific genes (Smith-Garvin et al., 2009). In the next section, we discuss WASp’s function in proximal TCR signaling.

### WASp’s Role in Proximal TCR Signaling

Assembly of WASp to SLP-76 signalosome upon TCR engagement is a key step for WASp to mediate the branching of the actin cytoskeleton polymerization (Wunderlich et al., 1999; Barda-Saad et al., 2005). Furthermore, two more pathways of WASp recruitment to distinct cellular compartments in the vicinity of TCR-CD3 have been reported and are the points to be discussed in this section.

**FIGURE 2:**
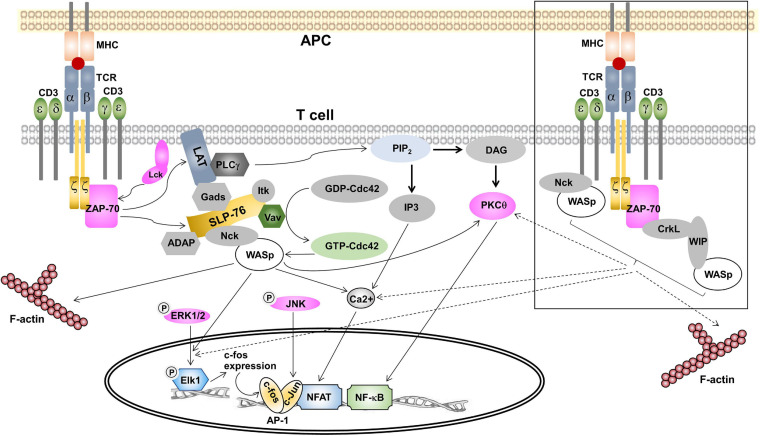
The role of WASp in signal transduction in T cells. TCR ligation with pMHC triggers signal transduction within the cytoplasm of T cells. Once phosphorylated by ZAP-70, phospho-LAT recruits proteins to form the LAT/SLP-76 signalosome containing Nck and Vav. Vav activates the Rho GTPase Cdc42, which in turn interacts with WASp. This complex is recruited to the signalosome *via* Nck. In association with GTP-Cdc42, WASp is released from the auto-inhibited conformation and then initiates the ARP2/3-dependent F-actin polymerization. In Ras/Rac-MAPK pathway, WASp is essential for the translocation of phospho-Erk1/2 into the nucleus. Once in the nucleus, phospho-Erk1/2 phosphorylates Elk1, which then causes c-fos expression, a component of transcription factor AP-1. WASp was not required for the activation of Jnk, a kinase causing c-jun expression that is the second component of AP-1. In the PKC pathway, WASp contributes to PKCθ activation in response to low doses of antigen. Activated PKCθ is required for activation of transcription factor NF-κB and its nuclear translocation. For Ca^2+^-mediated signaling, WASp is indispensable for intracellular Ca^2+^ mobilization, which is essential for NFAT translocation to the nucleus. In the squared box, two alternative pathways of WASp recruitment to TCR are shown. Firstly, WASp is constitutively associated with Nck and Nck-WASp is recruited to the TCR upon TCR ligation. Secondly, preformed CrkL-WIP-WASp is recruited to ZAP-70 following T cell activation. We propose that these two recent identified pools of WASp play a role in controlling actin polymerization and may contribute to the nuclear translocation of phospho-Erk1/2, PKCθ-mediated NF-κB activation and Ca^2+^ influx.

Ligand-binding to the TCR leads to the stabilization of the active conformation of the TCR (Gil et al., 2002; Minguet et al., 2007; Lee et al., 2015), in which the PRS, the RK motif and the ITAMs become exposed (Swamy et al., 2016; Hartl et al., 2020). In the active conformation the CD3ε PRS binds to the N-terminal SH3 domain of Nck, thus recruiting Nck to the TCR (Gil et al., 2002). Our recent work has suggested a role of actin polymerization in controlling Nck recruitment to CD3ε PRS (Wipa et al., 2020), although the exact mechanism remains enigmatic. At the same time Lck is recruited to the RK motif and the ITAMs can be phosphorylated (Hartl et al., 2020). Phosphorylation of CD3ε at the second ITAM tyrosine stabilizes the Nck-CD3ε interaction, since the SH2 domain of Nck can bind to the phospho-tyrosine (Paensuwan et al., 2016). This leaves the C-terminal SH3 domain of Nck free to interact with WASp. It has been found that WASp is constitutively associated with both Nck isoforms, Nck1 and Nck2, and both isoforms can associate with CD3ε (Gil et al., 2002). Indeed, we found that WASp can be recruited to the TCR upon TCR stimulation (Paensuwan et al., 2015; Figure 2), and this might be mediated by Nck. Thus, it was postulated that the direct recruitment of WASp to TCR-CD3 may also bring the effector molecules to TCR-CD3 that are essential for controlling the actin reorganization and signal transduction (Ngoenkam et al., 2018; Figure 2). Among these, GTP-Cdc42 has been found to be recruited to the T cell:APC contact site, where TCR, WASp and Vav-1 are accumulated (Cannon et al., 2001; Yokosuka and Saito, 2010). Localization of these proteins at the T cell:APC contact site supports the hypothesis of molecular machinery driven the actin polymerization at TCR. Further, recruitment of WASp to the TCR was relied on both the CD3 conformational change and partial CD3ε tyrosine phosphorylation (Paensuwan et al., 2015). This finding is coincident with the pattern of Nck recruitment to CD3ε following TCR triggering. This strengthens the possibility of Nck to mediate the recruitment of WASp to CD3ε. Further work is needed to assess the relative contributions of TCR-recruited WASp in regulating T cell activation. This finding may reveal the additional function of WASp besides its role in regulating actin polymerization such as controlling signal transduction in close proximity to the TCR.

Since WASp also binds to Lck (Matalon et al., 2013) recruitment of WASp to the TCR might help in phosphorylating the TCR. However, in WASp-deficient T cells phosphorylation of CD3ζ and ZAP-70 and total tyrosine proteins was undisturbed (Zhang et al., 1999), arguing against an important role of WASp in CD3 phosphorylation. It has been found in resting T cells that WASp is complexed with WIP (Ramesh et al., 1997), which directly interacts with the adaptor protein CrkL [CT10 regulator of kinase (Crk)-like] to form a CrkL-WIP-WASP complex. Following TCR ligation, CrkL, WIP and WASP were co-precipitated with ZAP-70 (Sasahara et al., 2002). Thus, a CrkL-WIP-WASP complex is recruited to ZAP-70 in response to TCR engagement to generate a ZAP-70-CrkL-WIP-WASp complex (Sasahara et al., 2002). Recruitment of this complex to the IS is mediated by the association of the CrkL SH2 domain to a phospho-tyrosine within interdomain B region of ZAP-70 (Chan et al., 1992). PKCθ then phosphorylates WIP thereby releasing WASp from WIP-mediated inhibition and then WASp is activated by membrane bound Cdc42 resulting in actin polymerization (Sasahara et al., 2002). Thus, besides *via* a SLP-76/Nck and a TCR/Nck complex, an alternative pathway of WASp recruitment to the IS is mediated by ZAP-70.

### WASp’s Role in the Ras/Rac-MAPK Pathway

In mammalian cells, there are three major members of MAPKs including Erk (Schaeffer and Weber, 1999), the p38 (Han and Ulevitch, 1999), and Jnk (Davis, 2000). Erk can be activated by Ras, while p38 and Jnk are activated by Rho family GTPases, including Rac and Cdc42 (Rincón et al., 2001). The nuclear target of these three MAP kinase members is the activation of the transcription factor AP-1.

T cells isolated from both WAS patient and WASp deficient (−/−) mice were unable to secrete IL-2 in response to TCR stimulation (Molina et al., 1993; Zhang et al., 1999; Cannon and Burkhardt, 2004). This impairment was not associated with TCR-proximal signaling since tyrosine phosphorylation of CD3ζ, ZAP-70, and total cellular protein detected in TCR-stimulated *WAS*^–^*^/^*^–^ murine T cells were similar to those observed in the wild-type counterparts (Zhang et al., 1999). The IL-2 promotor is activated by the transcription factors NFAT, AP-1 and NF-κB. The activity of AP-1 and expression of c-Fos, but not c-Jun were markedly impaired in T cells from WASp^–^*^/^*^–^ mice upon TCR stimulation (Cianferoni et al., 2005). Interestingly, upstream signaling proteins of AP-1 including Jnk, c-Jun, and Erk were normally phosphorylated. These results are consistent with previous studies showing that murine *WAS*^–^*^/^*^–^ T cells exhibited normal phosphorylation of Erk and Jnk (Zhang et al., 1999). However, translocation of phosphorylated Erk into the nucleus and phosphorylation of its nuclear substrate, Elk1 were impaired. This phosphorylated nuclear Elk is responsible for activation of c-Fos, a component of the transcription factor AP-1 (Cianferoni et al., 2005). Thus, WASp is essential only for nuclear translocation of phospho-Erk, Elk1 phosphorylation and expression of c-Fos.

### WASp’s Role in PKC-Mediated Signaling

T cells express different PKC isoforms, including PKC-α, δ, ϵ, η, θ, and ζ (Brezar et al., 2015). PKCθ is the most studied isoform and is the first PKC family member that is recruited to the IS (Arendt et al., 2002). PKCθ plays a key role in a range of signaling cascades that ultimately leads to activation of downstream transcription factors NF-κB, NFAT, and AP-1 (Baier-Bitterlich et al., 1996; Sun et al., 2000; [Bibr B49][Bibr B7]; Hayashi and Altman, 2007). In addition, PKCθ has been postulated to be involved TCR-induced actin polymerization through a process possibly mediated by WIP (Sasahara et al., 2002; Krzewski et al., 2006).

Once a ZAP-70-CrkL-WIP-WASp complex is formed at IS, PKCθ phosphorylates on WIP. This allows WASp to disengage from the WIP-WASp complex and to proceed its activation and function. As reported, PKCθ activation is dependent on Lck, Vav-1, ZAP-70, and SLP-76 (Sasahara et al., 2002). Thus, it has been proposed that the ZAP-70-CrkL-WIP pathway and PKCθ are the key players upstream of WASp activation (Sasahara et al., 2002). In keeping with this hypothesis, it was found that WASp-deficient T cells in response to antigen-specific APCs showed normal PKCθ polarization to the IS and stimulation of these cells with low doses of antigen caused diminished polarization of PKCθ (Cannon and Burkhardt, 2004), whereas activation with all peptide doses impaired IL-2 production in these cells. Thus, WASp is suggested to be the protein that can lower the threshold for organizing PKCθ at the IS. In addition, the roles of WASp on IS formation is proposed to be independent of its role in IL-2 production (Cannon and Burkhardt, 2004).

The data of WASp function in activation of NF-κB is scarce. However, it has been found that nuclear translocation and activity of NF-κB were normal in T cells from WASp^–/–^ mice, whereas NFAT dephosphorylation and nuclear localization, nuclear AP-1 binding activity, and expression of c-Fos were all impaired (Cianferoni et al., 2005). Moreover, in the T helper 1 (Th1)-skewed cells, mutation of the VCA domain that is required for Arp2/3-dependent F-actin polymerization did not affect NF-κB-p65 nuclear translocation (Sadhukhan et al., 2014). Together, WASp is required for PKCθ activation in response to low doses of antigen, but WASp is not required NF-κB activation in T cells.

### WASp’s Role in Ca^2+^-Mediated Signaling Pathways

T cell receptor engagement triggers the mobilization of Ca^2+^, which is required for T cell activation, gene expression, motility, synapse formation, cytotoxicity, development, and differentiation (Feske, 2007). Once entered into the cells, Ca^2+^ activates calcineurin resulting in nuclear translocation of NFAT (Gwack et al., 2007). Peripheral lymphocytes exclusively express NFAT-1, NFAT-2, and NFAT-4 (Amasaki et al., 1998). T cells from WAS patients and WASp-deficient mice show defects in IL-2 production upon TCR-induced T cell activation. This was associated with a partial reduction in intracellular Ca^2+^ mobilization compared with wild-type cells, while phosphorylation of CD3ζ, ZAP-70, and Erk was normal (Zhang et al., 1999; Badour et al., 2004; Cannon and Burkhardt, 2004). WASp is required for the assembly of the IS structure, which is essential for optimal sustained calcium signaling (Calvez et al., 2011).

Consistent with mouse WASp-deficient T cells, CD4^+^ and CD8^+^ T cells from WAS patients secrete low levels of IL-2 as well as IFN-γ, and TNF-α in response to stimulation with anti-CD3 and anti-CD28 antibody (Trifari et al., 2006). Defective cytokine production is associated with the reduction of nuclear translocation of NFAT-1 in CD4^+^ T cells, while a NFAT-1 and NFAT-2 reduction was observed in CD8^+^ T cells (Trifari et al., 2006). Previous reports show that the WH1 domain of WASp plays an important role in TCR-induced NFAT-mediated transcription (Silvin et al., 2001). It has been proposed that WASp WH1 possibly binds WIP to initiate transcriptional NFAT activation. In addition, mutation of WASp WH2 domain (also known as the verprolin domain), which is essential for Arp2/3-mediated actin polymerization, did not inhibit NFAT activation. Thus, it is suggested that WASp is indispensable for NFAT activation in a manner that is independent of its function in Arp2/3-induced actin polymerization (Silvin et al., 2001). WASp may indirectly regulate TCR-mediated NFAT activation through binding with adaptor protein Nck, which subsequently binds the Pak serine/threonine kinase to promote TCR-mediated NFAT activation (Yablonski et al., 1998).

## Conclusion Remarks

Since WASp has been identified, accumulating pieces of evidence reveal the role of WASp as a nucleation-promoting factor that transform signals from cell surface receptors to actin cytoskeleton rearrangement. Intense research has uncovered the functional domains of WASp as well as its interacting partners, most of which are involved in controlling actin-filament formation. Previous model of WASp recruitment to IS following TCR ligation is relied on SLP-76/Nck complex. However, two alternative pathways of WASp recruitment to IS were identified which are mediated by TCR-Nck and ZAP-70-CrkL-WIP. These findings reveal different WASp pool existing in T cells and which pool contributing to regulate actin polymerization is still opened for investigation. The essential role of WASp in actin-dependent T cell activation has been continually reported. However, little is known about WASp in TCR-mediated signal transduction and T cell activation. WASp-deficient cells show normal in TCR-proximal signaling such as phosphorylation of CD3ζ and ZAP-70. However, they show a defect in nuclear translocation of phospho-Erk and decreased intracellular Ca^2+^ mobilization, the exact cause of which is not known. Diminishing these signaling events results in an impairment of AP-1 and NFAT activation and reduced of cytokine production. WASp is not involved translocation of Jnk and NF-κB. Interestingly, independent roles of WASp in NFAT activation and actin polymerization have been proposed. Thus, understanding of the role played by WASp in TCR signaling is one of the recent scientific interests to improve the knowledge of etiology of WASp and the treatment of WAS.

## Author Contributions

JN collected the data and drafted the manuscript. PW and PP helped in retrieving the data. WS and SP supervised and edited the manuscript. All authors contributed to the article and approved the submitted version.

## Conflict of Interest

The authors declare that the research was conducted in the absence of any commercial or financial relationships that could be construed as a potential conflict of interest.
